# Metal-particle-induced enhancement of the photoluminescence from biomolecule-functionalized carbon nanotubes

**DOI:** 10.1186/1556-276X-9-85

**Published:** 2014-02-18

**Authors:** Se-Jin Kim, June Park, Yuhyun Jeong, Hayoung Go, Kangseok Lee, Seunghun Hong, Maeng-Je Seong

**Affiliations:** 1 Department of Physics, Chung-Ang University, Seoul 156-756, Republic of Korea; 2 Department of Life Science, Chung-Ang University, Seoul 156-756, Republic of Korea; 3 Department of Physics and Astronomy, Seoul National University, Seoul 156-757, Republic of Korea

**Keywords:** Carbon nanotube, Metal-particle-induced photoluminescence enhancement, RNA-functionalized carbon nanotube

## Abstract

The effect of metal particles on the photoluminescence (PL) and the Raman spectra of functionalized SWCNTs in aqueous solutions was systematically investigated by studying three different metal particles (gold, cobalt, and nickel) on three different SWCNT suspensions (DNA-, RNA-, and sodium deoxycholate salt (DOC)-functionalized SWCNTs). Substantial enhancement of the PL intensities was observed, while the Raman spectra remained unchanged, after gold, cobalt, or nickel particles were introduced into RNA-SWCNT aqueous suspensions. Almost the same results were obtained after the same metal particles were added to DNA-SWCNT aqueous suspensions. However, both the PL and the Raman spectra did not exhibit any change at all after the same metal particles were introduced into DOC-SWCNT aqueous suspensions. The unusual PL enhancements observed in this work cannot be accounted for by the three well-known mechanisms in the literature: surface-enhanced Raman scattering effect, Förster resonance energy transfer in a rebundling of isolated SWCNTs, and pH changes of the aqueous solutions.

## Background

Carbon nanotubes (CNTs) have been one of the most promising nanoscale materials for various applications due to their unique electrical, mechanical, thermal, and optical properties [1,2]. Nevertheless, bundling of CNTs, due to their strong hydrophobicity, is an obstacle for many applications. For biological applications of CNTs, making stable aqueous suspension of individual CNTs by functionalizing their surface with appropriate biomolecules is essential [3,4]. Single-stranded DNAs (ssDNAs) or double-stranded DNAs (dsDNAs) have been most commonly used for such functionalization of single-walled carbon nanotubes (SWCNTs), and optical properties of DNA-functionalized SWCNTs have been intensively studied [5-7].

Recently, SWCNT-based optical biosensors have been reported by several research groups [8-12]. Fluorescence bleaching of DAP-dex-functionalized SWCNTs when these complexes combine with nitric oxide was used for a nitric oxide (NO) sensor [8]. An avidin sensor application was demonstrated by showing a fluorescence recovery when DLC-functionalized SWCNTs combined with avidin [9]. The fluorescence quenching effect of insulin upon combining the insulin-binding-aptamer (IBA)-functionalized SWCNTs was used for an insulin detection [10]. Biosensor application using fluorescence recovery when molecular-beacon-DNA-functionalized SWCNTs combined with the conjugate DNA or thrombin was reported [11]. A Raman signal change of antibody-functionalized SWCNTs upon combining with corresponding bodies was demonstrated [12].

The optical property changes when metal ions or metal particles were introduced into a functionalized SWCNT suspension have also been extensively studied [13-18]. Photoluminescence (PL) enhancement of DNA-functionalized SWCNTs by terbium ions [13], fluorescence quenching of SDBS-functionalized SWCNTs by transition metal ions [14], fluorescence recovery of fluorophore-DNA-functionalized SWCNTs by silver ions and cysteine [15], and fluorescence quenching of GNQ-functionalized multi-walled carbon nanotubes (MWCNTs) by copper ions [16] were reported. Fluorescence quenching of PSMA-functionalized SWCNTs by gold nanoparticles of diameters of approximately 6 nm [17] was reported. But another study showed a Raman and fluorescence enhancement of SWCNTs by gold nanoparticles of diameters between 10 and 120 nm [18]. In spite of many previous reports, the effect of metal ions and metal particles on the optical property of functionalized SWCNTs is yet to be further investigated. In order to systematically study the effect of metal particles on the optical property of functionalized SWCNTs, we tried three different metal particles (gold, cobalt, and nickel) on three different SWCNT suspensions (DNA-, RNA-, and sodium deoxycholate salt (DOC)-functionalized SWCNTs). We observed substantial enhancement of PL signals from SWCNTs by all three metal particles in both DNA-SWCNT suspension and RNA-SWCNT suspension, but no change in PL by any metal particle was observed in DOC-SWCNT suspension.

## Methods

DNAs from herring sperm and DOC used in our work for functionalizing SWCNTs were purchased from Sigma-Aldrich (St. Louis, MO, USA). RNAs purified from *Escherichia coli* were obtained using the phenol extraction and ethanol precipitation method; and such as-purified total RNA dominantly consists of 2,904 (23S rRNA) and 1,542 (16S rRNA) nucleotides, corresponding to 990 and 480 nm in length, respectively. CoMoCAT SWCNTs were purchased from SouthWest Nanotechnologies Incorporated (Norman, OK, USA). The diameters of gold, cobalt, and nickel particles purchased from Alfa Aesar (Ward Hill, MA, USA) are 7.25 ± 1.75 μm, 1.40 ± 0.20 μm, and 5.00 ± 2.00 μm, respectively.

Aqueous suspensions of DNA-functionalized SWCNTs were prepared by adding SWCNTs (2.5 mg) to an aqueous DNA (0.68 mg/ml) solution of 25 ml, sonicating the solution using a bath-type sonicator (Branson 2510) for 2 h, and ultracentrifugation (T-1180; Kontron, Poway, CA, USA) at 50,000 × *g* for 1 h. Aqueous suspensions of RNA-functionalized SWCNTs were similarly prepared by adding SWCNTs (5 mg) to an aqueous RNA (1.4 mg/ml) solution of 50 ml, followed by the same sonication and centrifugation process. Aqueous suspensions of DOC-functionalized SWCNTs were prepared by adding SWCNTs (1 mg) to an aqueous DOC (2 wt.%) solution of 50 ml and sonicating the solution with a tip-type sonicator (Sonics Vibra cell VCX750; Sonics & Materials, Inc. Newtown, CT, USA) for 30 min, followed by the same centrifugation process.

Time-of-flight secondary ion mass spectrometry (TOF-SIMS) (TOF.SIMS^5^; ION-TOF, Heisenbergstr, Münster, Germany), with Bi^+^ as the primary ion source, was used to identify nucleotides in the synthesized DNA-SWCNT and RNA-SWCNT suspensions. PL and Raman spectra were measured at room temperature using 514 nm from an Ar^+^ laser (Innova 90C-6; Coherent Inc., Santa Clara, CA, USA) or 532-nm line from a frequency-doubled Nd:YAG laser (CL532-200-S; Crystalaser, Reno, Nevada, USA) as excitation light sources. Scattered light from the samples was analyzed through a single grating spectrometer (SP-2500i; Princeton Instruments, Trenton, NJ, USA) with a focal length of 50 cm and detected with a liquid-nitrogen-cooled silicon CCD detector (Princeton Instruments, Spec-10). A pH meter (Mettler Toledo, FE20; Thermo Fisher Scientific, Hudson, NH, USA) with glass electrodes was used to measure the pH of the solution samples.

In order to investigate the effect of metal particles on the PL and the Raman spectra, we carefully did as follows: 0.3 ml of the biomolecule-SWCNT suspension was put into a quartz cuvette, and PL and Raman spectra were taken right before 10 mg of each metal particle was added to the suspension; after a few minutes, most of the metal particles sedimented at the bottom of the cuvette; then PL and Raman spectra were taken at the exact same spot at 60, 120, and 180 min after the introduction of the metal particles.

## Results and discussion

**Figure 1:**
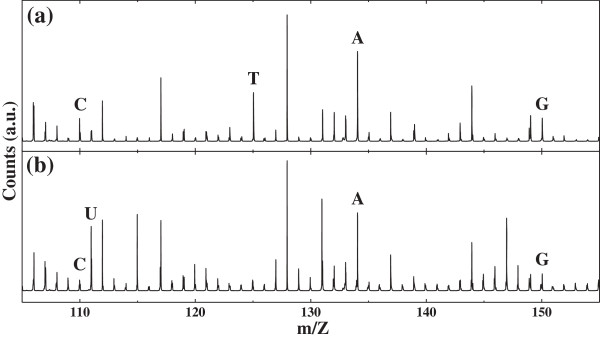
**Mass-to-charge-ratio spectra of the DNA- and RNA-functionalized SWCNTs measured by TOF-SIMS.** The DNA-functionalized SWCNTs shows four peaks C, T, A, and G **(a)** whereas the RNA-functionalized SWCNTs show four peaks C, U, A, and G **(b)**. The peak positions of the ionized nucleotides are as follows: C (C_4_H_4_N_3_O^-^, Cyt-H) at 110.03, U (C_4_H_3_N_2_O_2_^-^, Ura-H) at 111.02, T (C_5_H_5_N_2_O_2_^-^, Thy-H) at 125.03, A (C_5_H_4_N_5_^-^, Ade-H) 134.04, and G (C_5_H_4_N_5_O^-^, Gua-H) at 150.04.

**Figure 2:**
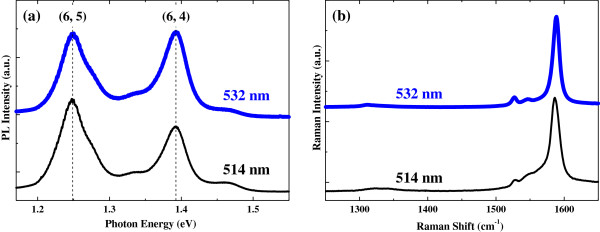
**Photoluminescence and Raman spectra of the RNA-functionalized SWCNTs.** Typical photoluminescence spectra **(a)** and typical Raman spectra **(b)** of our CoMoCAT SWCNTs functionalized with RNA for two different excitation lasers, 532 and 514 nm.

Before studying the effect of metal particles on the optical properties of DNA-SWCNT suspension and RNA-SWCNT suspension, we made sure that these suspensions were properly synthesized by doing TOF-SIMS, PL, and Raman measurements. TOF-SIMS can accurately identify five different nucleotides constituting DNA and RNA [19]. DNA consists of cytosine (cyt), thymine (thy), adenine (ade), and guanine (gua), whereas RNA consists of cytosine (cyt), uracil (ura), adenine (ade), and guanine (gua). Figure 1 shows the TOF-SIMS results of our DNA-functionalized SWCNTs (Figure 1a) and our RNA-functionalized SWCNTs (Figure 1b). The mass-to-charge-ratio peaks of the ionized nucleotides, nucleotides that are deprived of one proton, are clearly identified, indicating the existence of DNA and RNA in our DNA-SWCNT and RNA-SWCNT suspensions, respectively. Typical PL and Raman spectra of the RNA-functionalized SWCNTs are shown in Figure 2. Since we used CoMoCAT SWCNTs and the excitation laser wavelengths were 514 or 532 nm, the strong PL features observed at 1.25 and 1.39 eV were attributed to (6,5) and (6,4) nanotubes, respectively [20]. The 514- and 532-nm excitations resulted in almost the same PL and Raman spectra, apart from the slight differences in the relative PL intensity of (6,4) with respect to that of (6,5) and in the shoulder-like Raman feature on the low-frequency side of the G-band Raman signature at 1,587 cm^-1^ that can be attributed to a tiny difference in their resonant excitation conditions. It is worthy of note that the extremely weak signal intensity of the D-band near 1,350 cm^-1^ in Figure 2b indicates a very good structural quality of our SWCNTs.

**Figure 3:**
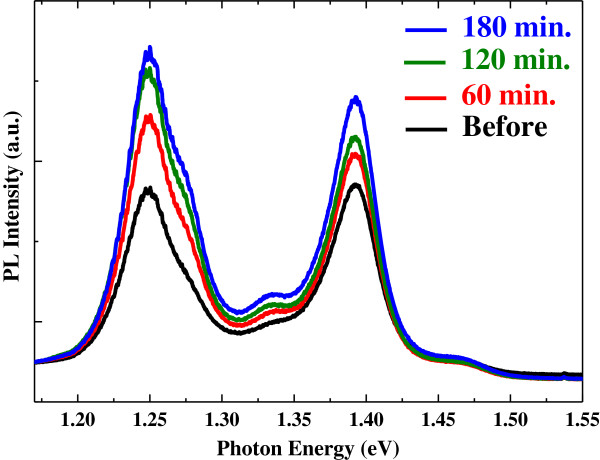
**Time evolution of the photoluminescence spectrum of the RNA-functionalized SWCNTs after adding Ni particles.** Excitation laser wavelength was 532 nm. The black spectrum was taken right before adding Ni particles, and the red, green, and blue spectra were taken 60, 120, and 180 min, respectively, after adding Ni particles.

Figure 3 shows a typical time evolution of the PL spectrum of the RNA-functionalized SWCNTs after Ni particles were added to the solution. All PL features exhibited concurrent enhancements. After 3 h or so, the observed PL enhancement was saturated and the PL intensity remained approximately Stable. A similar time evolution of the PL enhancements was observed for Au and Co particles in RNA-SWCNT solution and for Au, Ni, and Co particles in DNA-SWCNT solutions.

**Figure 4:**
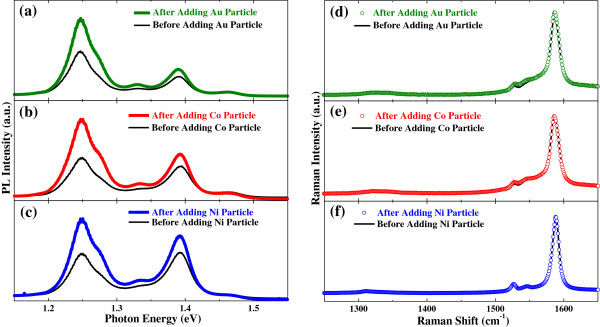
**Photoluminescence and Raman spectra of the RNA-functionalized SWCNTs before and after adding metal particles.** PL spectra show substantial enhancement after adding **(a)** gold, **(b)** cobalt, and **(c)** nickel particles. Raman spectra do not show any change after adding **(d)** gold, **(e)** cobalt, and **(f)** nickel particles. Excitation laser wavelength was 514 nm for **(a, b, d**, and **e)** and 532 nm for **(c** and **f)**. All the ‘after’ spectra were taken 180 min after adding metal particles.

Substantial PL enhancements in the aqueous RNA-SWCNT solution after metal particles were introduced can be seen in Figure 4a,b,c where PL spectra before and after the introduction of Au, Co, and Ni particles, respectively, were compared. However, the introduction of metal particles into the solution did not have any effect on the Raman spectrum as can be seen in Figure 4d,e,f.

**Figure 5:**
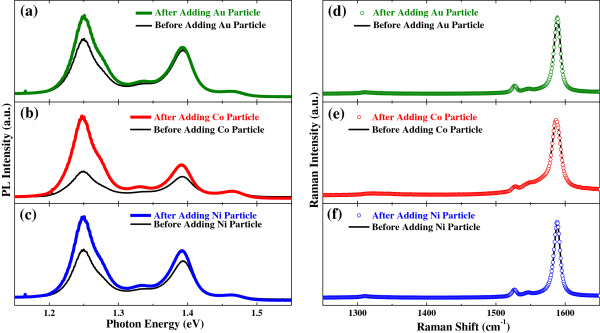
**Photoluminescence and Raman spectra of the DNA-functionalized SWCNTs before and after adding metal particles.** PL spectra show substantial enhancement after adding **(a)** gold, **(b)** cobalt, and **(c)** nickel particles. Raman spectra do not show any change after adding **(d)** gold, **(e)** cobalt, and **(f)** nickel particles. Excitation laser wavelength was 532 nm for **(a, c, d, and f)** and 514 nm for **(b and e)**. All the ‘after’ spectra were taken 180 min after adding metal particles.

**Figure 6:**
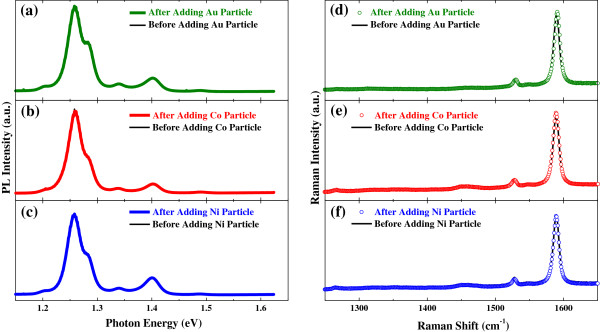
**Photoluminescence and Raman spectra of the DOC-functionalized SWCNTs before and after adding metal particles.** Both Raman spectra do not show any change after adding **(a and d)** gold, **(b and e)** cobalt, and **(c and f)** nickel particles. Excitation laser wavelength was 532 nm for all spectra. All the ‘after’ spectra were taken 180 min after adding metal particles.

In order to see that the observed metal-particle-induced PL enhancement is a unique phenomenon for the RNA-functionalized SWCNTs, we performed the same experiments on the DNA-functionalized SWCNTs. The results, as shown in Figure 5, are almost the same as those on the RNA-functionalized SWCNTs. Finally, we did the same experiments on the DOC-functionalized SWCNTs. However, the PL spectrum as well as the Raman spectrum remained unchanged after the metal particles were introduced into the DOC-SWCNT solution, as shown in Figure 6.

The atomic force microscopy (AFM) results (see Additional file 1) showed that the metal particles were not adsorbed on the SWCNTs. In fact, the size of the metal particles is a few micrometers whereas the diameter of the SWCNTs is approximately 1 nm. Thus, the metal particles are too big to be adsorbed on the SWCNTs. The metal particles just sedimented at the bottom of the cuvette and remained there during the optical measurements. Thus, the probed biomolecule-SWCNT suspension inside the laser spot was not directly in contact with the metal particles.

There are three well-known mechanisms for SWCNT PL enhancement. Surface-enhanced Raman scattering (SERS) effect is known to enhance PL intensities as well as Raman intensities via an amplified electric field near metal particles or a metal surface [21-23]. Since the Raman intensities of our sample did not show any enhancement at all, in spite of substantial PL enhancement, SERS effect cannot explain our PL enhancement results. PL enhancement, via Förster resonance energy transfer (FRET), was reported when a rebundling of isolated SWCNTs occurred, where the PL enhancement was accompanied by a peak red-shift or a suppression of high-energy PL peak intensity [20,24-26]. There was no PL peak shift, and all the PL features were enhanced concurrently in our results. Thus, we can rule out FRET as the underlying mechanism of our PL enhancements. It is well known that pH has a strong effect on PL intensity of SWCNTs. At low pH environment, the surface oxidation of SWCNTs causes a PL bleaching, but the PL intensity recovers at high pH [27-29]. We have measured the pH change before and after the introduction of metal particles, and the measured pH increases were less than 0.3 for all three metal particles, Au, Co, and Ni, which is too small to induce any observable PL enhancement. Nonetheless, it is worthwhile to note that oxygen desorption from SWCNTs results in a PL enhancement [29]. Thus, it would be reasonable to assert that oxygen desorption occurred for the biomolecule-functionalized SWCNTs upon the introduction of metal particles into the biomolecule-SWCNT suspension whereas it did not for the DOC-functionalized SWCNTs. Biomolecules such as DNA and RNA are structurally more flexible than the inorganic surfactant DOC. Subtle changes of the solution induced by metal particles, e.g., slight pH change, could make biomolecules highly susceptible to some structural change, which could lead to oxygen desorption from SWCNTs.

## Conclusions

In summary, we have systematically investigated the effect of metal particles on the PL and the Raman spectra of functionalized SWCNTs in aqueous solutions. Substantial enhancement of the PL intensities was observed, while the Raman spectra remained unchanged, after gold, cobalt, or nickel particles were introduced into RNA-SWCNT aqueous suspensions. Almost the same results were obtained after the same metal particles were added to DNA-SWCNT aqueous suspensions. However, both the PL and the Raman spectra did not exhibit any change at all after the same metal particles were introduced into DOC-SWCNT aqueous suspensions. The unusual PL enhancements observed in this work cannot be accounted for by the three well-known mechanisms in the literature; SERS effect, FRET in a rebundling of isolated SWCNTs, and pH changes of the aqueous solutions. We suggest that oxygen desorption which occurred in the biomolecule-functionalized SWCNTs upon the introduction of metal particles be responsible for the observed PL enhancement.

## Abbreviations

DAP-Dex: 3,4-diaminophenyl-functionalized dextran; DLC: dye-ligand conjugate; DOC: sodium deoxycholate salt; IBA: insulin-binding-aptamer; GNQ: glycine-*N*-8-quinolylamide; PL: photoluminescence; SDBS: sodium dodecylbenzensulfonate; SWCNT: single-walled carbon nanotube; TOF-SIMS: time-of-flight secondary ion mass spectrometry.

## Competing interests

The authors declare that they have no competing interests.

## Authors’ contributions

SK, JP, and YJ carried out the experiments. HG and KL prepared RNA and DNA samples. SK and MS analyzed the data and drafted the manuscript. MS initiated and supervised the work. SK, KL, and MS contributed to discussing, reviewing, and editing the manuscript before submission. SH provided the AFM results. All authors read and approved the final manuscript.

## Supplementary Material

Additional file 1 AFM images showing the morphology of SWCNTs. Click here for file
