# Supplementary material for: Metal-particle-induced enhancement of the photoluminescence from biomolecule-functionalized carbon nanotubes
Source: Nanoscale Res Lett. 2014 Feb 18;9(1):85. doi: 10.1186/1556-276X-9-85 (PMC3931836; doi:10.1186/1556-276X-9-85)
Supplement: Additional file 1 — AFM images showing the morphology of SWCNTs. [file 1556-276X-9-85-S1.docx]

**Additional file 1:**

Metal-particle-induced enhancement of the photoluminescence from biomolecule-functionalized carbon nanotubes

Se-Jin Kim^1^, June Park^1^, Yuhyun Jeong^1^, Hayoung Go^2^, Kangseok Lee^2^, Seunghun Hong^3^, Maeng-Je Seong^[[1]](#footnote-1)^*

**Morphology of SWCNTs**

Atomic force microscopy (AFM) measurements were performed in order to investigate the morphology of SWCNTs before and after the introduction of Co particles into the DNA-functionalized SWCNT suspensions. The results are shown in Figs. S1 and S2. The height of the DNA-SWCNT composite is approximately 1 nm ± 0.3 nm. Thus, the tube-like features in the AFM images in Figs. S1 and S2 can be attributed to isolated individual SWCNTs, functionalized with DNA, considering that the average nanotube diameter of our SWCNTs is ~ 0.8 nm and that the thickness of DNA is ~ 0.3 nm. The size of the Co particles is a few μm and they are too big to be adsorbed to SWCNTs. Thus, one would expect that the morphology of SWCNT-DNA remains almost unchanged after the Co particles were introduced into the SWCNT-DNA suspensions, as illustrated in the AFM images and the height profiles in Figs. S1 and S2.


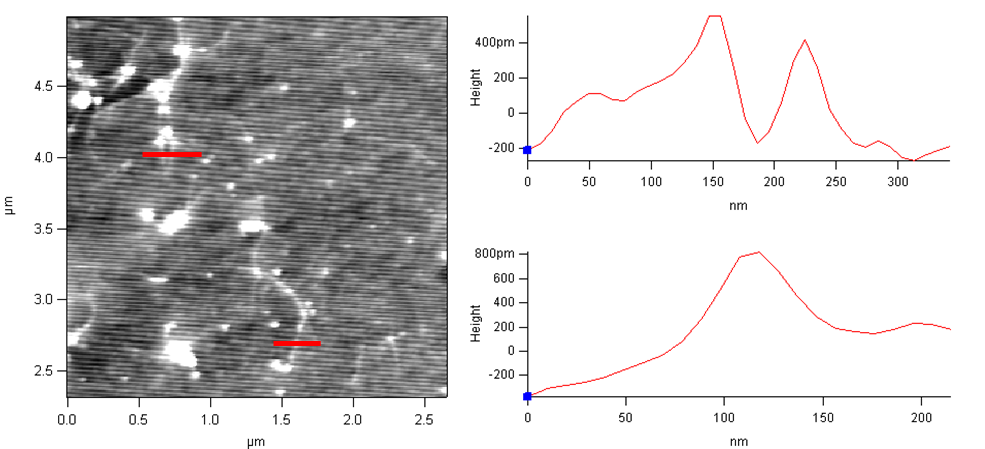


Figure S1 AFM image and height profiles of the DNA-functionalized SWCNTs on a silicon substrate


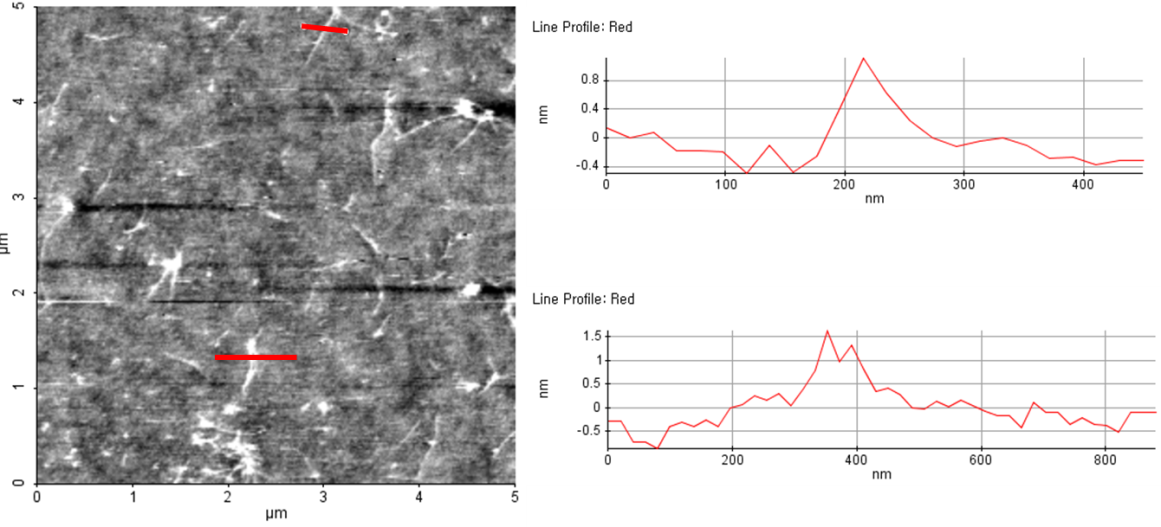


Figure S2 AFM image and height profiles of the DNA-functionalized SWCNTs, after the introduction of Co particles, on a silicon substrate

1. * Correspondence: mseong@cau.ac.kr

   ^1^Department of physics, Chung-Ang University, Seoul 156-756, Republic of Korea

   ^2^Department of Life Science, Chung-Ang University, Seoul 156-756, Republic of Korea

   ^3^Department of physics and Astronomy, Seoul National University, Seoul 151-747, Republic of Korea [↑](#footnote-ref-1)
